# Forest Sound Classification Dataset: FSC22

**DOI:** 10.3390/s23042032

**Published:** 2023-02-10

**Authors:** Meelan Bandara, Roshinie Jayasundara, Isuru Ariyarathne, Dulani Meedeniya, Charith Perera

**Affiliations:** 1 Department of Computer Science & Engineering, University of Moratuwa, Moratuwa 10400, Sri Lanka; 2 School of Computer Science and Informatics, Cardiff University, Cardiff CF24 3AA, UK

**Keywords:** forest acoustic dataset, environment sound classification, machine learning, Freesound, deep learning

## Abstract

The study of environmental sound classification (ESC) has become popular over the years due to the intricate nature of environmental sounds and the evolution of deep learning (DL) techniques. Forest ESC is one use case of ESC, which has been widely experimented with recently to identify illegal activities inside a forest. However, at present, there is a limitation of public datasets specific to all the possible sounds in a forest environment. Most of the existing experiments have been done using generic environment sound datasets such as ESC-50, U8K, and FSD50K. Importantly, in DL-based sound classification, the lack of quality data can cause misguided information, and the predictions obtained remain questionable. Hence, there is a requirement for a well-defined benchmark forest environment sound dataset. This paper proposes FSC22, which fills the gap of a benchmark dataset for forest environmental sound classification. It includes 2025 sound clips under 27 acoustic classes, which contain possible sounds in a forest environment. We discuss the procedure of dataset preparation and validate it through different baseline sound classification models. Additionally, it provides an analysis of the new dataset compared to other available datasets. Therefore, this dataset can be used by researchers and developers who are working on forest observatory tasks.

## 1. Introduction

Environmental sound recognition is a widely used technique when identifying various sound events for surveillance or monitoring systems based on the acoustic environment. Several investigations have been carried out with different techniques in the context of a forest monitoring system to protect forest reserves. For example, prior studies have experimented with different sound classification approaches for the recognition of various species and possible forest threats such as illegal logging, poaching, and wildfire [1,2,3,4,5]. In such systems, environmental sounds are captured, processed using a modelling algorithm, and classified into different sound classes.

With technical advancements, sound classification approaches evolved from machine learning (ML) models such as K-nearest neighbours (KNN) [3,6,7], XGBoost [8,9], Gaussian mixture modelling (GMM) [5,10], and support vector machine (SVM) [6,11,12] to deep learning. Deep neural networks (DNNs) such as convolutional neural networks (CNN) and recurrent neural networks (RNN) require a large quantity of labelled data compared to ML for a promising result. Hence, when using DL-based approaches, a well-biased and rich dataset with a relatively large data size is essential as the performance keeps increasing with a quality dataset.

Although several studies have been carried out in the forest acoustic monitoring context, still, a standard benchmark dataset specific to forest sounds is unavailable. Therefore, most of the existing studies have utilized publicly available environmental sound datasets such as ESC-50 [4,13,14,15,16,17], UrbanSound8K (U8k) [14,18,19,20,21], FSD50K [22,23], and SONYC-UST [24,25]. These datasets contain a large quantity of audio data categorized into several groups covering a broad area of sound events. However, a limited number of classes can be used for forest environment sound classification, and most data are irrelevant for such a domain. Since a significant number of resources need to be utilized to extract data from datasets and to annotate the data points according to a suitable taxonomy, the direct use of public datasets for the classification model is ineffective.

Additionally, some studies have utilized datasets such as BIRDZ [26,27] and xeno-canto Archive [28,29,30], which contain only bird sounds. The xeno-canto archive is an open audio collection dedicated to sharing bird sounds, and BIRDZ is a control audio dataset originating from the xeno-canto archive, which contains a subset of 11 bird species. As it contains audio data specific to one class, several such datasets need to be used in the forest sound classification system. Moreover, several researchers have experimented with private datasets due to the unavailability of forest-specific sound datasets. For instance, in such studies, they have deployed sound sensors in a forest environment and recorded the sound events to create a dataset according to their requirements [6,31,32]. In contrast, some studies have created datasets using audio clips collected from online sound data repositories such as Freesound [3,5,11]. With a closer look at the literature, it can be identified that the forest acoustic monitoring domain suffers from certain shortcomings including a lack of a standard taxonomy and the unavailability of a public benchmark dataset. These limitations motivated us to introduce a new dataset for the domain. Accordingly, the novelty of this paper is to present a standard dataset for forest sound classification and to provide a comprehensive overview of the procedure for creating and validating the dataset. Addressing the current research gaps, we introduce FSC22 [33], a novel benchmark dataset for the acoustic-based forest monitoring domain. It contains 2025 5 s long audio clips originating from an online audio database Freesound. All sound events are categorized into 6 major classes, which are further divided into 34 subclasses. For the initial phase of dataset composition, 27 subclasses were picked, and 75 audio samples were collected per class. Each audio clip was manually annotated and verified to ensure the quality of the dataset. The key contributions of this paper can be summarized as follows.

- Introduces a novel public benchmark dataset consisting of forest environmental sounds, which can be utilized for acoustic-based forest monitoring.
- Presents a comprehensive description of the methodology used for dataset creation, including data acquisition from Freesound, filtering, validation, and normalization.
- Explains the baseline models used for the sound classification and the selection criteria for those models.
- Provides a detailed evaluation of the dataset using human classification, ML-based, and DL-based classification.
- Presents a comprehensive discussion of the results obtained with the proposed FSC22 dataset and compares them with the publicly available datasets.

We have created the FSC22 dataset and made it freely available to support and motivate future researchers in this domain [33]. We expect that this dataset will help research communities to better understand forest acoustic surveillance and experiment with the domain. The rest of the paper is structured as follows. Section 2 explores the related datasets used in previous research. Section 3 provides an overview of the taxonomy of the proposed dataset. Section 4 introduces the FSC22 dataset, including the data collection methodology and its importance to the acoustic domain. Section 5 provides a comprehensive description of the baseline-model-based dataset evaluation approach. Section 6 describes the experiments conducted on the dataset namely human classification and baseline-model-based classification, with the results and observations. Finally, Section 7 concludes the paper.

## 2. Related Work

Seminal contributions have been made to the ESC context in recent years. Among those, several instances of research carried out for forest acoustic monitoring can be identified. Forest acoustic monitoring is crucial as it provides a firm basis of evidence to arrive at conclusions to conserve forest coverage and species. However, due to the unavailability of a comprehensive forest-specific sound dataset, most of the previous research on forest monitoring was done using a common environmental sound dataset or a private dataset according to the requirement. This section provides an overview of the publicly available environmentally sound datasets and other datasets utilized by previous researchers in this domain. However, to the best of our knowledge, there is no forest-specific sound dataset in the literature.

**Figure 1:**
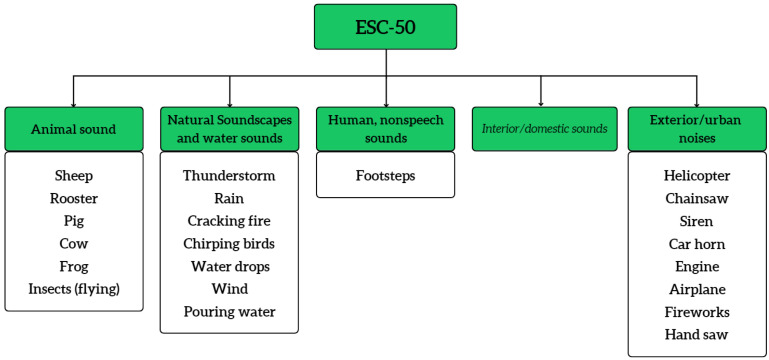
ESC-50 dataset.

**Figure 2:**
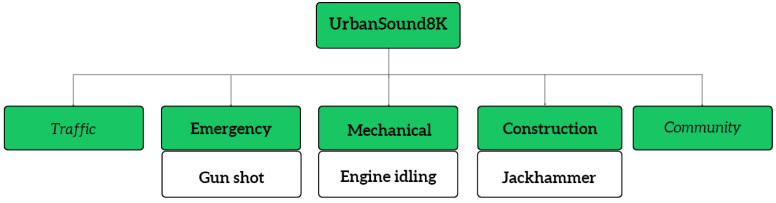
Urbansound8K dataset.

Among the available datasets, ESC-50 [34] is a frequently used environmental sound dataset for forest acoustic monitoring. For instance, Andreadis et al. [4] utilized ESC-50 to detect illegal tree-cutting and identify animal species. ESC-50 is a dataset consisting of 2000 environmental audio clips under 50 classes of common sound events. It contains 40 5 s long recording samples per class, extracted from Freesound. Figure 1 shows a section of the ESC-50 dataset taxonomy emphasizing forest-specific sounds. Moreover, U8K [35] is another popular dataset used in many types of research on audio-based monitoring systems [18,36]. U8K is a subset of the main Urban Sound dataset, which contains 8732 labelled sound clips of urban sounds from 10 classes. The classes of this dataset were drawn from the urban sound taxonomy [37], and all the recordings were extracted from Freesound. Figure 2 includes a part of the U8K dataset taxonomy mostly relevant to the forest environment sound domain. FSD50K [38] is an open dataset of human-labelled sound events. It consists of over 51K audio clips totalling over 100 h of audio manually labelled using 200 classes. The classes of this dataset are drawn from the AudioSet ontology [39]. The three above-mentioned datasets were created using the audio extracted from the Freesound project. It is an audio-based public dataset that contains more than 500,000 audio clips.

**Figure 3:**
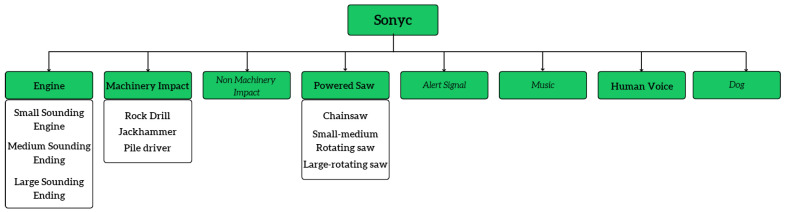
SONYC-UST-V2 dataset.

Moreover, SONYC-UST [40] is another quality dataset, where data are grouped into 8 main classes and further divided into 23 fine-grained classes. This can be considered a more realistic dataset as it was created using the audio data acquired using acoustic sensors deployed in New York City. Figure 3 shows a part of the SONYC-UST dataset taxonomy highlighting the audio classes specific to forest monitoring and surveillance. AudioSet [41] is another audio event dataset, including over 2M tracks from Youtube videos. Every 10 s video is annotated using over 500 sound classes derived from the AudioSet ontology [39]. The main concern with AudioSet is it cannot be considered an open dataset due to the copyright issues and terms of services constraint from Youtube. In addition, as the clips were collected from Youtube, they may consist of clips with poor quality and can disappear after a certain time due to privacy issues or copyright claims. Table 1 presents a summary of the existing environmental sound datasets.

Additionally, several other domain-specific dataset usages were reported in prior studies on environment sound observatory systems. For bird sound identification studies, the xeno-canto archive [43], which is a bird-sound-sharing portal, was used to acquire the audio data essential for the experiment [28,30,44]. BIRDZ dataset, which is a real-world audio dataset made using the xeno-canto archive was also used in the related literature [45,46]. Similarly, the usage of the BirdCLEF dataset was identified in prior studies, which consists of 62,902 audio files and is publicly available on Kaggle [47]. As all these datasets are specific to a certain sound class, a combination of several such datasets is required when developing a complete forest monitoring system.

Many researchers have experimented with a private dataset created according to their requirements, due to the scarcity of forest-specific sound datasets. Such datasets are generated using the audio data acquired from online sound repositories or audio recorded by acoustic sensors or as a combination of both. Mporas et al. [3] created a chainsaw sound dataset, including the background noises such as rain and wind, using the sounds acquired from freely available sound repositories. Ying et al. [11] experimented with an animal sound recognition system, and the required animal sounds were acquired from Freesound. In contrast, Assoukpou et al. [6] combined the chainsaw sounds recorded from acoustic sensors deployed in three different forest areas and other sounds acquired from online websites to create a dataset to identify chainsaw sounds.

Accordingly, many environmental-sound classification studies have utilized the datasets mentioned above with different sound classification approaches. In most of the studies, CNN models were widely adopted as a firm basis for prominent audio classification models [20,36,48]. Moreover, there are instances where ML algorithms were utilized for audio classification [49]. One of the key distinctions when choosing between DL and ML was the availability of well-labelled and high volumes of data. DL algorithms scale with the data while increasing the performance, whereas ML plateaus at a certain level of performance when adding more data. Table 2 shows an overview of DL and ML approaches deployed for sound classification using the ESC-50 and U8K datasets.

## 3. FSC22 Taxonomy

Prominent research efforts carried out in the forest acoustic classification domain have been based on a subset of an already established public dataset such as ESC50, U8K, or on small self-made datasets. Thus, the requirement for a well-defined dataset dedicated to forest acoustics can be identified. As the first step of creating a benchmark dataset, a standard taxonomy that can showcase and capture all the different acoustic scenarios present in forest ecosystems needs to be established.

**Figure 4:**
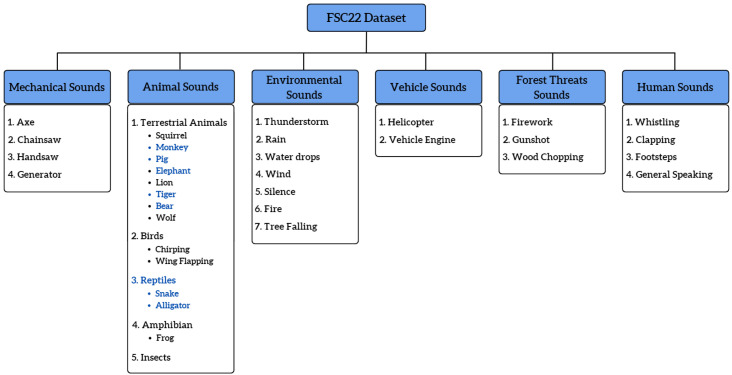
FSC22 taxonomy.

In the parent level of the proposed taxonomy, all the acoustic scenarios were classified into six classes: mechanical sounds, animal sounds, environmental sounds, vehicle sounds, forest threat sounds, and human sounds. Further, each class was divided into subclasses that captured specific sounds which fell under the main category. For example, under the main class, mechanical sounds, four subclasses were identified, namely axe, chainsaw, handsaw, and generator. This subdivision aimed to introduce specific class labels to prevent the usage of generalized labels such as tree cutting, animal roar, etc. Figure 4 presents the complete forest sound taxonomy developed as a base for the creation of the FSC22 dataset. Further, it showcases the complete subdivision of the main 6 classes into 34 subclasses. We selected only 27 subclasses for the FSC22 dataset ignoring 7 subclasses shown in blue colour, due to the unavailability of a sufficient number of sound clips in Freesound. Though all the left-out classes had more than 200 search results in the Freesound platform, most of the audio clips were artificially generated or included unnecessary noise making them unsuitable to be included in the FSC22 dataset.

The proposed taxonomy aimed at covering two main objectives. The first objective was to completely cover fundamental acoustic scenarios such as chainsaw sounds, tree felling, and wildfire, which are extensively used for research works. The second objective was to provide high-quality, normalized audio under unambiguous class labels. We extensively analysed the related literature that utilized forest acoustics and identified the most essential and frequent types of acoustic phenomenon that should be available in a benchmark dataset to fulfil the first objective, as explained in Section 4. It should be noted that the proposed taxonomy is not fixed and with time, more related acoustic classes under forest acoustics need to be added while refining the taxonomy to achieve saturation.

## 4. FSC22 Dataset

**Figure 5:**
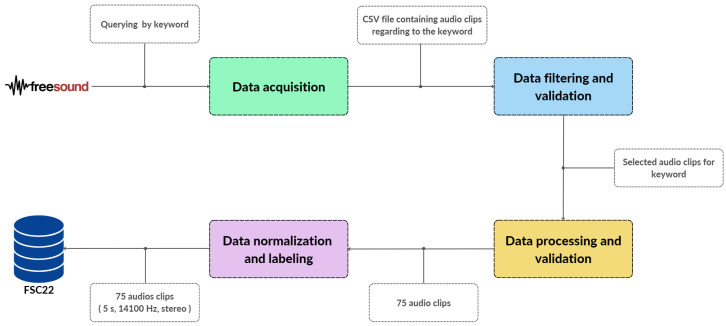
Overall procedure.

The proposed FSC22 dataset [33] in this paper is a public benchmark dataset containing 2025 audio samples normalized to a 44,100 Hz sample rate, 16-bit depth, and a stereo channel configuration. All the audio samples were distributed between six major parent-level classes. Each audio was further divided into scenario-specific low-level classes, which captured the context of the considered audio sample as described in Section 3. The FSC22 dataset serves two major objectives, the first one being the requirement to provide sufficient audio samples for widely researched forest-related acoustic classes. The second objective is to present high-quality normalized audio samples under event-specific class labels. This section describes the procedure which was followed to develop the FSC22 dataset while ensuring the objectives. Figure 5 shows the overall procedure of creating the FSC22 dataset and each subprocess is described in this section.

### 4.1. Dataset Preparation

#### 4.1.1. Data Acquisition

The development of major datasets governing the acoustic classification domain is mainly based on online audio collection portals such as YouTube, BBC Sound Effects Library, and Freesound. The usage of such sources presents unique advantages and disadvantages. Therefore, it was initially required to select the source that FSC22 would be based upon, to develop a high-quality benchmark dataset. Although both YouTube and BBC Sound Effects Library are rich when some acoustic labels are considered, they publicly present copyright issues when publishing the final dataset. Freesound, available at https://freesound.org/, (accessed on 27 September 2022), is a free, public, online platform where thousands of audio data are published, and it was identified that by basing the content of FSC22 on the Freesound platform, we could easily navigate the publishing issue. Further, the API endpoints available on the Freesound platform allow users to write python scripts to search for different audio scenarios and download the metadata and the corresponding audio files without manually searching and downloading the audio.

**Figure 6:**
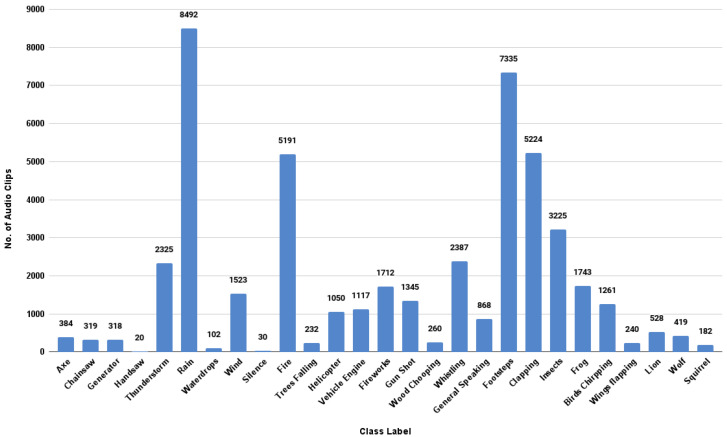
The number of audio samples per class.

As the first step of data acquisition, we selected 27 classes from the FSC22 taxonomy to complete in the first phase of the FSC22 dataset. For each of the selected class labels, we queried audio samples, which contained the considered label in the title or the description, using the API endpoint for a text search. The querying process was completed through a python script. After all the matching audio samples were identified, their metadata were written classwise to spreadsheet files to be fed to the filtering and validation stage. We selected 47,832 audio clips and sent them for the filtering and validation step. Figure 6 showcases the number of audio samples identified via the data-acquiring step for each selected 27 class.

#### 4.1.2. Data Filtering and Validation Phase

**Figure 7:**
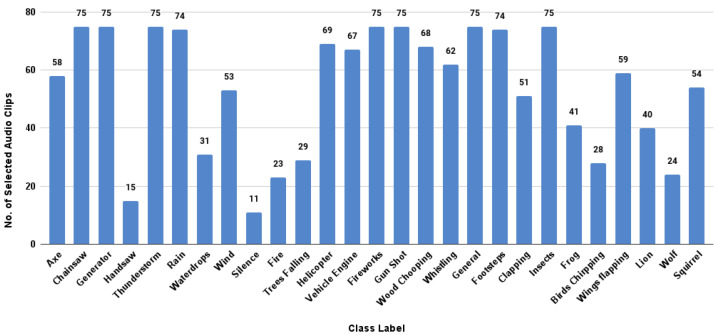
The number of selected audio samples per class.

After the spreadsheet files were completed for all the selected classes, all the sheets were traversed to remove unsuitable query results which were present in the sheets due to the noise associated with the API endpoint. After the filtering of suitable audio samples was completed, each selected audio sample was manually checked by listening to them and downloaded for further processing to begin. All the unclear or unsuitable audio samples for further processing were removed to refine the dataset quality. Figure 7 shows the number of audio samples selected from each class to be further processed to complete the FSC22 dataset.

#### 4.1.3. Data Processing and Validation

In order to generate 75 audio clips for each audio class, the downloaded audio samples were processed based on the duration of the original file. Audacity software was used for this procedure, which is an open-source application for audio editing and tagging. The downloaded audio files were uploaded to the Audacity application and trimmed to 5 s. Selected audio samples with longer duration were spliced into multiple recordings of 5 s. This step was necessary for some classes due to the lack of suitable audio samples to complete the considered audio sample limit per class. This process was repeated for all the sound classes, and 75 audio recordings were validated and finalized at the end.

#### 4.1.4. Data Normalization and Labelling

After the filtering and validation process was completed, all 27 classes selected for the first phase were finalized with 75 audio recordings. As the first step of normalization, the sampling frequency was set to 44,100 Hz, the bit depth was set to 16, and the channel setting was configured to stereo for all the selected audio recordings, using the load function of Librosa. In the audio extraction step, from the original audio samples in the earlier phase, audio samples with nearly 5 s of duration were extracted. Hence, as the second step of normalization, the duration of all the selected audio samples was set to 5 s by trimming excess parts or by padding with silence accordingly.

At the end of the normalization process, all the original audio samples were renamed accordingly. In this step, the source file name was mapped into the dataset file name in the format of UniqueClassIndex_UniqueAudioID.wav. The first part of the label indicates the class related to the audio sample and is followed by a unique audio ID. Proper labelling of the audio files makes it easier to navigate through the dataset. Once the audio files were labelled, the corresponding metadata were entered into the base metadata file to complete the development of the FSC22 dataset.

### 4.2. Content Description

FSC22 is a public benchmark dataset that can be utilized in research work governing forest acoustic monitoring and classification. The dataset was developed according to the taxonomy proposed in Section 3. Out of the 34 subclasses listed in the taxonomy, 27 subclasses were completed for the first phase of the FSC22 dataset. Each subclass contains 75 selected audio samples, which have been manually checked for any inconsistencies. Overall, the dataset contains 2025 audio samples, each with a duration of 5 s, resulting in 2.81 h of forest acoustics under the specified class labels. All the required information about the audio samples available in the dataset is listed under the metadata file located in the FSC22 master folder. The FSC22 master folder contains two subfolders, audio wise V1.0 which includes the 2025 audio samples, and the metadata folder which holds the Metadata.csv file.

Readers of this study and the users of the FSC22 dataset should note that each audio sample was renamed according to the following convention to better support the usage of the new dataset.

- UniqueClassIndex_UniqueAudioID.wav eg: 1_10101.wav

Table 3 provides a snapshot of the Metadata.csv file for the convenience of the readers. As shown for each audio file, the metadata file provides:

- Source file name—ID of the original audio sample, used to extract the corresponding audio sample.
- Dataset file name—ID of the audio, in the context of FSC22.
- Class ID—class identification index (an integer from the range 1 to 27).
- Class name—class name in which the audio sample is classified.

### 4.3. Importance of FSC22 for the Forest Acoustics Domain

Analysing the research contributions made towards the forest acoustic domain, it becomes evident that a publicly available forest-specific sound dataset is unattainable. Due to the scarcity of a standard dataset for forest sounds, the research community has experimented with different approaches for data acquisition. Few can be identified as obtaining sound recordings by employing sound sensors, collecting sound clips available in online sound repositories, and extracting the sounds from YouTube videos. Table 4 summarizes the sound acquisition approaches used in previous forest acoustic domain research for a better overview.

The findings in Table 4 confirmed that in most of the early studies, authors prepared a separate dataset according to their requirements due to the unavailability of a proper forest acoustic dataset. However, data collection is a complex and time-consuming task which could be an overhead for research tasks. Hence, the requirement for a standard dataset arises. Addressing the problem of the unavailability of a standard dataset, this paper introduces FSC22, which includes forest-specific sounds under 27 classes. This dataset covers most of the general acoustic classes identified in a forest environment. The FSC22 dataset will be a great contribution to any further research performed under the forest acoustic domain.

## 5. Methods and Technical Implementation

For ESC, both ML and DL have been extensively used in the related literature. Therefore, we provide classification experiments covering both architectures. An extreme gradient boosting (XGBoost)-based experiment is provided for the ML approach, while a CNN-based experiment is provided for the DL approach. These models were used as the baseline models.

### 5.1. Feature Engineering

**Figure 8:**
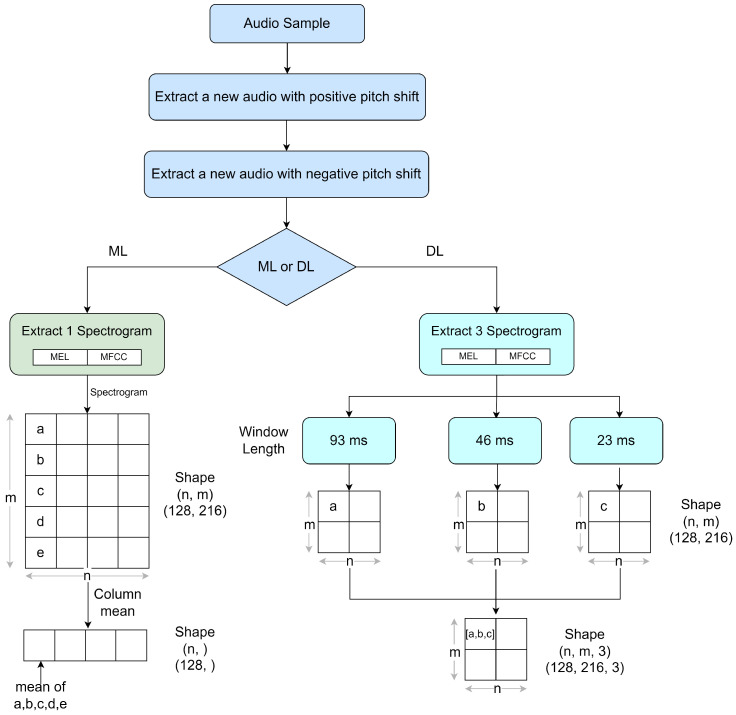
Feature preparation methodology.

Feature engineering is a principal requirement for a successful ML pipeline. Studies focusing on the audio classification domain properly emphasize the requirement of advanced feature-engineering techniques such as the usage of spectrograms to represent audio samples in the time and frequency domains [4,6,10,17,52] and audio augmentation techniques to prevent overfitting of the prediction algorithm [13,14,46,53,54] to obtain state-of-the-art classification performances. This section provides an overview of the feature-engineering techniques followed in the proposed experiments as shown in Figure 8.

#### 5.1.1. Considered Datasets

As described in Section 4.3, quality audio data are scarce in the forest acoustics domain; thus, a benchmark dataset that could be used to compare the quality of the proposed FSC22 dataset could not be identified in the related literature. The ESC50 dataset, which is a benchmark dataset used in the ESC domain, was therefore used to compare the performance of the FSC22 dataset. For the study, 2000 audio recordings, each of 5 s duration, distributed into 50 unique classes from the ESC50 dataset, and 2025 audio recordings each of 5 s duration distributed into 27 unique classes from the FSC22 dataset were selected.

#### 5.1.2. Data Augmentation Technique

Data augmentation is an important step in the feature engineering phase to artificially expand the available data samples for training and testing ML and DL algorithms. Especially when it comes to DL approaches, models suffer from overfitting when the quantity of training data available is considerably less [55]. For the proposed experiments, positive pitch shifting and negative pitch shifting, where the pitch of audio recordings is increased and decreased by two steps, respectively, were utilised [56]. The pitch shifting was implemented with the pitch_shift function provided by the Librosa.effects library for python.

As a result of a single audio sample, two new augmented audio samples were created increasing the quantity of data available. In summary, due to the augmentation with pitch shift, the number of audio samples from ESC50 was increased to 6000, while the FSC22 dataset increased to 6025 audio samples. For both datasets, 80% of the audio samples were used for training the model, while 20% were used for validating the performance of the trained model, by following the Pareto principle as in most of the general cases, 80% of the effects come from 20% of the causes.

#### 5.1.3. Feature Extraction

In the audio classification domain, the general practice is to us spectrograms, representing an audio signal in both time and frequency domains, as the feature extraction mechanism. The mel spectrogram (MEL) [20,57] and the mel frequency cepstral coefficients (MFCC) [3,10], which can be identified as the two most utilized spectrograms, were used to extract the features for this study. In order to extract the spectrograms from the raw audio data, the mel spectrogram and MFCC were provided by the librosa.feature library. Using both functions, each audio file was sampled into overlapping frames, and for each frame model coefficient or mel frequency, cepstral coefficients were calculated. Thus, the calculated coefficients were returned as a two-dimensional array of shapes (number of coefficients × number of samples). As a further improvement, for the mel spectrograms obtained, all the coefficients were converted to the decibel scale from the power scale.

As shown in Figure 8, ML-based classification techniques generally utilize one-dimensional features. Therefore, it was required to reduce the dimensionality of the created spectrograms, before they were used with the XGBoost model. This was achieved by aggregating the one-dimensional feature vectors extracted for each overlapping frame into a single vector by taking their mean value. For the DL-based classification, an image-like representation of the features according to the RGB mode was required. Hence, for each audio sample, three spectrograms were created by changing the length of the window used for framing. The created spectrograms had a windowing length of 93 ms, 46 ms, and 23 ms, and this was achieved by keeping the sample rate parameter at 22,050 Hz and the n_fft parameter at 2048, 1024, and 512, respectively.

### 5.2. Machine-Learning-Based Classification

The related literature exploring the automated classification of acoustic phenomena that are abundant in forest ecosystems has utilized different ML algorithms to carry out the classification task. Among such efforts, ML algorithms such as KNN, SVM, and random forests can commonly be identified. Due to the superiority of the extreme gradient boosting (XGBoost) algorithm against such traditional ML algorithms, this study explored the usability of XGBoost to properly classify forest acoustics.

XGBoost is capable of handling nonlinear relationships in the features. Handling nonlinear relationships are important in sound classification as there are many nonlinear relationships between the sound features and the class labels. Moreover, XGBoost has the ability to learn from the errors made by previous trees. Additionally, XGBoost use L1 and L2 regularizations, which is important to reduce overfittings.

The XGBoost library available for python was used to conduct the tests and the model parameters were used to fine-tune the performance of the implemented model. As the final set of parameters, num_class was set to 27, the multiclass classification error rate was used as the eval_metric, subsample, colsample_bytree and min_child_weight were set to 1, max_depth was 6, learning_rate was 0.3, and 100 n_estimators were used. Further, to improve the memory efficiency and the training speed of the XGBoost model, both the training and validation datasets were converted to the internal data structure (DMatrix) used by the model, which was optimized for both memory efficiency and training speed. Then, the configured model was trained with 80% of the considered dataset, and the evaluation was completed with the remaining 20% of the data using the trained XGBoost model.

### 5.3. CNN-Based Classification

Although it can be identified that a substantial number of studies have used ML-based algorithms to classify unstructured data such as audio and images, DL-based models can outperform the traditional ML models with considerable margins, due to their ability to extract features from raw data [58]. For the study, a convolutional neural network-based [14,59] model consisting of 9 layers was utilized, based on the work of the authors of [36].

**Figure 9:**
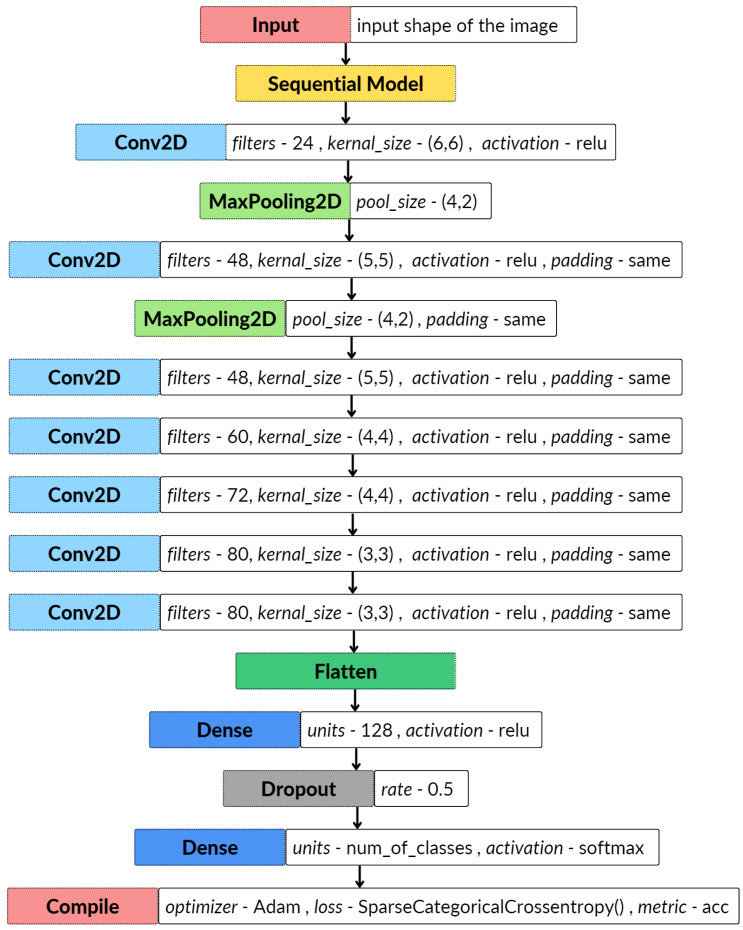
The CNN based architecture of the model.

Similarly, as in the ML-based approach, 80% of the data were used to train and fine-tune the CNN model, while the remaining 20% was used for the validation procedure. The model was configured to run for 50 epochs; however, an early stopping callback function was used to stop the model from overfitting to the training data. The implementation of the model was completed using the Keras library provided by TensorFlow [60]. Figure 9 presents the architecture of the model accompanied by the parameters used to implement the model using the Keras library.

## 6. Dataset Evaluation

In order to analyse the performance and characteristics of the FSC22 dataset, three major classification experiments were performed. As the first phase, a human classification experiment was conducted to identify a baseline classification accuracy for the FSC22 dataset. An ML- and DL-based classification of the FSC22 dataset was conducted as the second phase, to generate comparable performance scores with respect to related studies. Finally, the same ML and DL models were tested with the ESC50 dataset to present the general performance of the developed models. This section describes each experiment and the results obtained.

### 6.1. Human Classification Result of FSC22

Hearing and identifying sound through the human auditory system depend on a series of complex steps. Scientists have discovered that a form of auditory learning occurs in daily life to help us identify and memorize sound patterns. Hence, when a certain sound pattern differs from a small factor such as a noisy background, humans find it difficult to recognize the exact sound type. Humans’ identification of sound types comes with a high level of uncertainty, which may differ from machine classification. In order to identify this difference in human decisions, a human classification experiment was carried out for the created dataset. For this experiment, 25 participants in the age group 20–30 were selected. The survey included audio-based questions where the participants were instructed to select the correct label after listening to the sound clips [61]. For the creation of the survey, the Free Online Survey Software and Tools|The QuestionPro^®^ platform [62] was used. The questionnaire contained two randomly selected audio clips from each class and altogether 54 questions were included for the 27 classes. For each question, four choices of labels were given.

**Figure 10:**
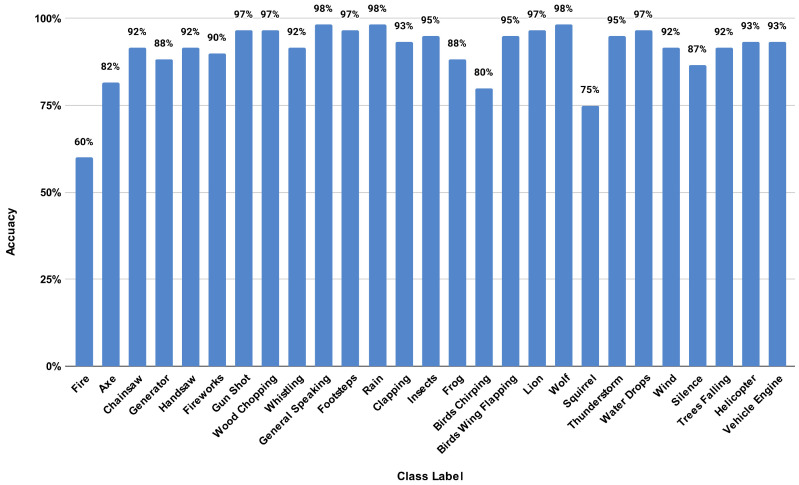
Class accuracies obtained in human classification.

After the completion of the survey, an overall accuracy of 91% was observed for the selected audio samples. These survey responses were used to calculate the class-level recognition accuracies. It was identified that the human candidates achieved a maximum classification accuracy of 98% for the classes, Wolf, General Speaking, and Rain, while the two classes, Squirrel and Fire, achieved the lowest accuracies, showing the hardness to identify such sounds by the human auditory system. Figure 10 shows the human classification accuracies obtained for all the classes of the FSC22 dataset.

### 6.2. Baseline-Model-Based Classification Results of FSC22

As the second approach for our dataset evaluation, a baseline classification analysis was performed using XGBoost- and CNN-based models. Section 5 provides a detailed overview of the baseline model selection and classification procedure. With the desired target accuracy results obtained through human classification in Section 6.1, the next goal was to investigate the level of performance that could be achieved on a baseline model classification.

The baseline XGBoost- and CNN-based models were evaluated on the FSC22 dataset using the evaluation metrics accuracy, F1-score, precision, and recall. Accuracy is the most intuitive performance measure, and it provides the ratio of the correctly predicted samples to the total samples, while precision provides the ratio of correctly predicted positive samples to the total predicted positive samples, and recall gives the ratio of correctly predicted positive samples to all samples in the actual class. The F1-Score is the weighted average of precision and recall. The metrics module of the Scikit-learn (Sklearn) library was used to calculate all the metrics; for the precision, recall, and F1-score, averaging was done using the unweighted mean as all the classes were balanced for both datasets. Table 5 and Table 6 provide the summary of results obtained by evaluating the FSC22 dataset against the baseline XGBoost- and CNN-based models, respectively.

#### 6.2.1. Results of ML-Based Classification

**Figure 11:**
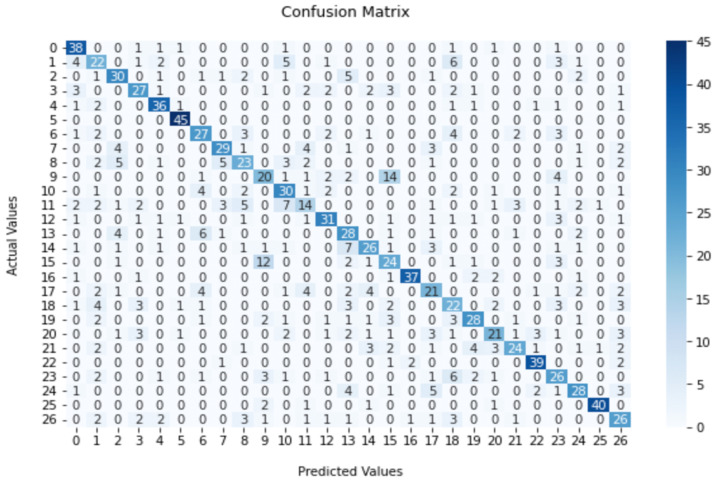
Confusion matrix for XGBoost-based classification with MFCC and augmented data.

As shown in Table 5, the FSC22 dataset had an average classification accuracy ranging from 48.14% to 62.17% for the selected XGBoost ML model. The highest classification accuracy of 62.71% was reported for the model with the MFCC feature extraction mechanism. In order to better analyse the results, the confusion matrix of the approach with the highest reported accuracy is displayed in Figure 11. A confusion matrix visualizes and summarizes the performance of a classification algorithm. According to the matrix, it can be identified that the Silence and Bird Chirping classes obtained the highest-class level accuracy of 99.58% and 98.84%, respectively. Moreover, the Axe class and Generator class showed the lowest accuracies among the 27 classes.

#### 6.2.2. Results of CNN-Based Classification

**Figure 12:**
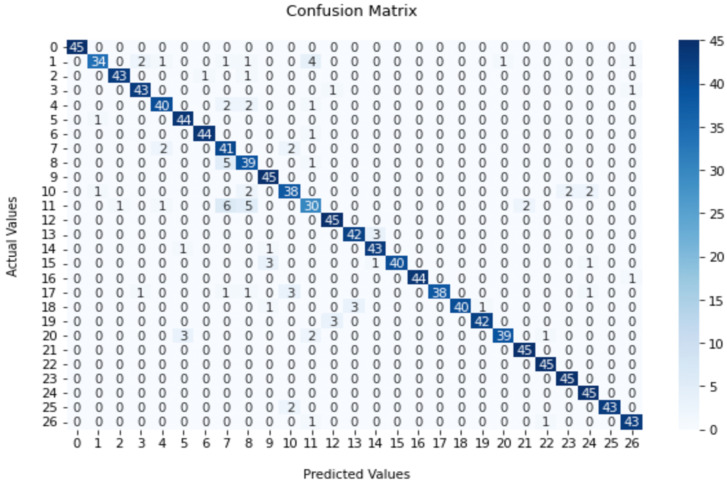
Confusion matrix for CNN-based classification with mel spectrogram and augmented data.

When compared with the ML-based classification approach, the CNN-based classification showed a significant performance with the FSC22 dataset. As reported in Table 6, the dataset had an average classification accuracy ranging from 53.08% to 92.59% for the CNN model. Out of the four classification accuracies, 92.59% was the highest, which was obtained for the CNN model with the MEL feature extraction mechanism. The confusion matrix given in Figure 12 for the approach which had the highest overall accuracy was used to evaluate the class-level accuracy of the dataset. According to the matrix, it is apparent that almost all the classes had a very high accuracy level, while Generator and Rain classes obtained the lowest among them.

### 6.3. Model Evaluation Results of the ESC-50 Dataset

All the trials conducted with the two feature extraction approaches for the ML- and CNN-based classification of the FSC22 dataset were tested with the ESC50 dataset. All the conducted experiments were evaluated based on the metrics presented in Section 6.2. Table 7 showcases the results obtained with the ML approach, while Table 8 presents the CNN-based classification results. It can be identified that the trial which used data augmentation and the MFCC feature extraction obtained the highest accuracy of 53.25% for the ML-based approach. Moreover, the CNN-based approach which used the mel-spectrogram-based feature extraction supported with data augmentation generated the highest classification accuracy of 92.16%.

## 7. Discussion

### 7.1. Lessons Learned

We conducted eight performance comparisons over the FSC22 dataset, as shown in Table 5 and Table 6. These experiments are listed as follows.

- E1: accuracy of XGBoost model with MFCC and data augmentation;
- E2: accuracy of XGBoost model with MFCC and no data augmentation;
- E3: accuracy of XGBoost model with mel spectrogram and data augmentation;
- E4: accuracy of XGBoost model with mel spectrogram and no data augmentation);
- E5: accuracy of CNN model with MFCC and data augmentation;
- E6: accuracy of CNN model with MFCC and no data augmentation;
- E7: accuracy of CNN model with mel spectrogram and data augmentation;
- E8: accuracy of CNN model with mel spectrogram and no data augmentation.

The same experiments were conducted over the ESC50 dataset to further support the observations as shown in Table 7 and Table 8. This section provides a discussion of the observations made after the experiments were completed.

#### 7.1.1. ML vs. DL for Environmental Sound Classification

ML and DL techniques have been extensively used in the related literature for environmental sound classification. To establish a performance comparison between ML and DL architectures over the FSC22 and ESC50 datasets, eight comparisons were made based on the above-defined performance measures. For FSC22, the CNN model outperformed the XGBoost model by a significant margin for all the comparisons, E1 vs. E5, E2 vs. E6, E3 vs. E7 and E4 vs. E8. For the ESC50 dataset, the CNN-based approach outperformed the XGBoost approach in the comparisons E1 vs. E5, E3 vs. E7 and E4 vs. E8. The XGBoost outperformed the CNN model when MFCC was used for the feature extraction of the nonaugmented dataset (E2 vs. E6). A careful evaluation of results published in the related literature provides similar evidence, identifying that DL algorithms perform better when it comes to complex classification tasks such as audio data tagging. It can be identified that this is due to reasons such as the ability of DL algorithms to extract inherent features from the raw data avoiding selective invariance [55], the ability of DL algorithms to learn from large volumes of data [36], and less requirement for feature engineering before the training of the model. Although DL approaches present higher accuracies compared to ML ones, they need high resources for the training to complete, and the resulting models are complex and suffer from low interpretability and explainability [63]. Thus, for a proper real-world deployment of a DL-based sound classification system, further research is required to understand and improve the underlying dynamics.

#### 7.1.2. Importance of Data Augmentation Techniques

A major requirement to develop proper artificial intelligence models is the availability of large volumes of quality data. When the forest sound classification domain is considered, the availability of well-defined, quality public data is limited. Although the proposed FSC22 dataset provided 2025 audio recordings providing 2.81 h of recording time, the data volume was not sufficient to properly train a CNN, RNN, or ML model to achieve state-of-the-art results. Data augmentation techniques can be successfully used to expand the available data points and to present the significance. As observable by the results of Table 7 and Table 8, the performance of the XGBoost- and CNN-based models showed a significant improvement in accuracy when augmentation techniques were employed, compared to the performance obtained without augmentation. The CNN model used with the FSC22 dataset showed accuracy degradations of 40% and 43% for the MFCC-based and mel-spectrogram-based approaches, respectively, when data augmentation was not applied. Similarly, the XGBoost model showed decrements of 12% and 14% for the two feature extraction approaches, MFCC and mel spectrogram, respectively. Accuracy reductions could be identified for the tests conducted with the ESC50 dataset as well. This empirical evidence showcases the importance of using data augmentation techniques when training artificial intelligence algorithms. Although we successfully implemented baseline data augmentation techniques to increase model performance, further research is required to understand novel techniques that can solve data insufficiency issues while preventing models from overfitting.

#### 7.1.3. Feature Representation Methodology

In the domain of audio classification, extracting feature embeddings that can accurately represent the audio signal is of the utmost importance. For the ML and DL models implemented in this study, mel spectrograms and MFCC spectrograms were employed as discussed in Section 5.1.3. With the experiments conducted for both FSC22 and ESC50 datasets using the ML-based approach, it could be identified that the usage of an MFCC-based feature extraction outperformed the tests conducted with mel spectrograms as the feature representation. However, for the DL-based approach, a mel-spectrogram-based feature extraction provided the highest accuracies, except for the test conducted without augmentation for the FSC22 dataset. Hence, in the context of this study, a clear separation cannot be drawn between the two spectrogram methods, for the task of representing audio signals.

### 7.2. Comparison with Existing Sound Datasets

Due to the unavailability of a publicly available benchmark dataset to be used for forest acoustic classification tasks, researchers have utilized different techniques to fulfil their data requirements as explained in Section 4.3. Table 9 provides a comparison between the results of existing studies and the highest-performing approach proposed in this paper. Accordingly, it can be seen that this study utilized the highest number of audio recordings distributed in 27 unique forest acoustic classes while achieving state-of-the-art classification accuracies for the forest-sound-classification-based studies. However, when the model performance is compared to the state-of-the-art performances achieved for the broader ESC domain, it can be identified that the results published in this paper require further refinement. Therefore, as future directions, applying transfer learning using the ImageNet dataset [36,64,65], exploring different data augmentation techniques [32,46,66] and feature representation methodologies [67,68] are suggested by the authors.

### 7.3. Future Research Directions

This study introduced the FSC22 dataset and proposed a baseline architecture for the classification of forest acoustics. As presented in Section 7.2, the developed CNN-based classification model outperformed existing forest acoustics classifier systems. We identify the following directions for the reference of researchers working in the forest acoustics domain.

#### 7.3.1. Practical Deployment of Forest Acoustic Classification Systems

Forest acoustic classification systems can provide valuable information to protect forest reserves from natural and artificial phenomena. A practical implementation will require the classification model to be deployed in a resource-constrained edge device, which will be challenging. The best-performing CNN model proposed in this study contains 4.6 million parameters and to be deployed in an edge device, complexity needs to be reduced substantially. Techniques such as pruning, XNOR-NET, and bottleneck layers can be effectively used to reduce the model complexities but will reduce the model performance by a significant amount [41]. Hence, future work is required to identify methodologies to generate reduced complexity models for FSC while preserving the classification accuracy on a reasonable scale.

#### 7.3.2. Explainability and Interpretability of FSC Models

Explainability and the interpretability of machine learning models is an emerging domain which presents interesting effects on the way that ML models are utilised. Explainability refers to the ability of a learning model to provide human-understandable explanations for its predictions. Interpretability refers to the ability to understand the internal workings of a model and how it arrives at its predictions. Forest sound classification systems with the potential of being deployed in the forest ecosystems to help authorities can greatly benefit from a transparent classification model. The number of studies covering the explainability and the interpretability of ESC or FSC models is scarce. Thus, we recommend future researchers working in this domain to contribute to develop more explainable and interpretable audio classification models.

Apart from the above two major research directions, it can be identified that state-of-the-art ESC models have comparatively high performance measures with respect to the identified FSC models including the CNN-based model proposed in this study. State-of-the-art ESC models utilise techniques such as transfer learning from the Imagenet dataset, multiple aggregated feature representations, multiple data augmentation strategies to achieve very high performance measures. Therefore, the authors recommend future researchers working on FSC to explore such techniques and utilise them to improve the performance measures of current FSC models to a comparable scale.

## 8. Conclusions

Environment sound classification (ESC) using artificial intelligence is a prominent research area in audio recognition. Under ESC, forest sound classification (FSC), which focuses on identifying artificial and natural phenomena observable in forest ecosystems, has received a high amount of research interest. The recognition of forest sounds generates highly valuable use cases in scenarios such as illegal logging, poaching, and wildfires. FSC suffers from the unavailability of a standard sound taxonomy and the unavailability of a sufficiently large public benchmark dataset. With the intention of resolving both issues, this study presented the FSC22 taxonomy and the first version of the FSC22 dataset. The first version of the FSC22 dataset consisted of 2025 human-annotated, 5 s long audio recordings equally distributed into 27 unique classes. The authors intend to expand the first version of the FSC22 dataset in the future, capturing more acoustic classes according to the FSC22 taxonomy. Further, the study presented CNN-based and XGBoost-based classification experiments using the FSC22 dataset. The CNN-based approach achieved a maximum classification accuracy of 92.59%, while the XGBoost model achieved a maximum accuracy of 62.71%. A survey conducted with 25 human candidates to identify different sounds from the classes listed in the FSC22 dataset was also conducted to establish a baseline accuracy score. Finally, the authors believe that the proposed FSC22 taxonomy, the created FSC22 V1.0 dataset, the experiments conducted, and the discussions provided through this study will support future research work governing the FSC domain.

## Figures and Tables

**Table 1:** Summary of existing ESC datasets.

Dataset	Source	Total Clips	Clip Length	Classes
ESC-50 [34]	Freesound	2000	5 s	50
Urban-Sound8K [35]	Freesound	8732	More than 4 s	10
AudioSet [41]	Youtube	2M	10 s	527
FSD50K [42]	Freesound	51,197	0.3 s to 30 s	200
SONYC- UST-v2 [40]	SONYC acoustic	18,510	10 s	23

**Table 2:** Related studies on sound classification using ESC-50 and UrbanSound8K Dataset.

Study	ML/DL	ESC-50		UrbanSound8K	
		Model	Accuracy	Model	Accuracy
[20]	DL(CNN)	DenseNet	98.50%	DenseNet	97.10%
		AlexNet	88.10%	AlexNet	93%
		ResNet	96.80%	ResNet	99.20%
[36]	DL(CNN)	DenseNet	97.57%	DenseNet	99.20%
		ResNet	96.80%	ResNet	99.49%
[48]	DL(CNN)	DenseNet	92.80%	DenseNet	87.40%
[50]	ML			SVM	71%

**Table 3:** Sample of metadata of the FSC22 dataset.

Source File Name	Dataset File Name	Class ID	Class Name
17548__A.wav	1_10101.wav	1	Fire
17548__B.wav	1_10102.wav	1	Fire
17548__C.wav	1_10103.wav	1	Fire

**Table 4:** Sound acquisition approaches in related studies.

Study	Domain	Source	Dataset Acquiring Approach
[3]	Illegal logging detection	Freely available online sound data repositories	Collected audio recordings of chainsaws and environment background noises (rain, wind, birds)
[11]	Animal sound recognition	Freesound	Collected bird sounds, mammal sounds, and insect sounds
[10]	Tree-cutting detection	Sensor recordings from an urban environment	Collected 18 chainsaw sounds, 27 vehicle sounds, 20 forest-specific sounds, 28 background sound clips
[51]	Animal sound recognition	HU-ASA database	Collected 1418 animal sound clips from the archive
[44]	Bird species detection	xeno-canto	Collected 2104 sound clips for 5 bird species
[4]	Illegal tree cutting	ESC-50	Selected 7 specific classes related to forest environment (wind, chainsaw, rain, birds, etc.)
[6]	Chainsaw sound identification	Sensor recordings from a forest environment and online sound repositories	Collected 301 chainsaw sounds and 2964 other sounds (bird, insects, animals, etc.)
[5]	Chainsaw and vehicle sound detection	Sensor recordings from forest and urban environments	Acquired 57 chainsaw recordings, 70 vehicle/engine sounds, 62 forest sounds, 28 general urban sounds
[31]	Illegal logging detection	Sensor recordings from a forest environment	Collected 100 chainsaw sounds

**Table 5:** Results of ML-based classification of the FSC22 dataset.

Feature Representation	Augmentation	Accuracy	F1-Score	Precision	Recall
MFCC	Applied	62.71%	0.62	0.63	0.62
MFCC	Not applied	55.06%	0.54	0.55	0.55
Mel spectrogram	Applied	56.04%	0.56	0.57	0.56
Mel spectrogram	Not applied	48.14%	0.47	0.48	0.48

**Table 6:** Results of CNN-based classification of the FSC22 dataset.

Feature Representation	Augmentation	Accuracy	F1-Score	Precision	Recall
MFCC	Applied	89.30%	0.893	0.898	0.893
MFCC	Not applied	53.82%	0.533	0.552	0.538
Mel spectrogram	Applied	92.59%	0.925	0.929	0.925
Mel spectrogram	Not applied	53.08%	0.52	0.53	0.53

**Table 7:** Results of ML-based classification of ESC50 dataset.

Feature Representation	Augmentation	Accuracy	F1-Score	Precision	Recall
MFCC	Applied	53.25%	0.525	0.529	0.532
MFCC	Not applied	43.50%	0.431	0.455	0.435
Mel spectrogram	Applied	48.18%	0.478	0.493	0.481
Mel spectrogram	Not applied	31.75%	0.309	0.325	0.317

**Table 8:** Results of CNN-based classification of ESC50 dataset.

Feature Representation	Augmentation	Accuracy	F1-Score	Precision	Recall
MFCC	Applied	85.41%	0.855	0.866	0.854
MFCC	Not applied	42.25%	0.408	0.443	0.422
Mel spectrogram	Applied	92.16%	0.921	0.925	0.921
Mel spectrogram	Not applied	44.75%	0.429	0.448	0.447

**Table 9:** Comparison of existing datasets.

Paper	Model	Quantity of Data	Types of Data Used	Feature	Metric	Result
[3]	SVM	Total duration of around 5 min	Chainsaw sounds with background noise	MFCC	Accuracy	91.07%
[11]	Random forest	40	Bird sounds, mammal sounds, insect sounds from Freesound	Double features	Average accuracy rates in different environments (rain, wind, traffic, average)	86.28%
[51]	Cyclic HMM	1418	Animal sounds from HU-ASA database	MFCC	Accuracy	64%
[4]	Configuration based on a CNN	280	Chainsaw sounds, chirping birds, crackling fire, crickets, handsaw, rain, and wind extracted from ESC50	MFCC	Accuracy	85.37%
[6]	SVM with log kernel	3265	Chainsaw sounds	MFCC	TPR	53.16%
[5]	Feed-forward network	217	Chainsaw sounds, vehicle/engine sounds, forest sounds, urban sounds	Fourier power spectrum coefficients	Accuracy	79.50%
[31]	CNN	100	Chainsaw	Fourier spectrogram	Accuracy	96%
**This Study**	CNN	2025	27 unique classes	Mel spectrogram	Accuracy	92.59%

## Data Availability

“FSC22 Dataset”, IEEE Dataport, doi: https://dx.doi.org/10.21227/40ds-0z76.
